# Network modules uncover mechanisms of skeletal muscle dysfunction in COPD patients

**DOI:** 10.1186/s12967-018-1405-y

**Published:** 2018-02-20

**Authors:** Ákos Tényi, Isaac Cano, Francesco Marabita, Narsis Kiani, Susana G. Kalko, Esther Barreiro, Pedro de Atauri, Marta Cascante, David Gomez-Cabrero, Josep Roca

**Affiliations:** 1 Hospital Clinic de Barcelona, Institut d’Investigacions Biomèdiques August Pi i Sunyer (IDIBAPS), Universitat de Barcelona, Barcelona, Spain; 2 Center for Biomedical Network Research in Respiratory Diseases (CIBERES), Madrid, Spain; 3 Unit of Computational Medicine, Department of Medicine, Karolinska Institute, 171 77 Stockholm, Sweden; 4 Center for Molecular Medicine, Karolinska Institutet, 171 77 Stockholm, Sweden; 5 Bioinformatics Core Facility, IDIBAPS-CEK, Hospital Clínic, University de Barcelona, Barcelona, Spain; 6 Pulmonology Dept, Muscle and Respiratory System Research Unit, IMIM-Hospital del Mar, Universitat Pompeu Fabra, PRBB, Barcelona, Spain; 7 Departament de Bioquimica i Biologia Molecular, Facultat de Biologia-IBUB, Universitat de Barcelona, 08028 Barcelona, Spain; 8 Mucosal and Salivary Biology Division, King’s College London Dental Institute, London, SE1 9RT UK

**Keywords:** Gene modules, Chronic obstructive pulmonary disease, Exercise training, Systems medicine, Muscular weakness

## Abstract

**Background:**

Chronic obstructive pulmonary disease (COPD) patients often show skeletal muscle dysfunction that has a prominent negative impact on prognosis. The study aims to further explore underlying mechanisms of skeletal muscle dysfunction as a characteristic systemic effect of COPD, potentially modifiable with preventive interventions (i.e. muscle training). The research analyzes network module associated pathways and evaluates the findings using independent measurements.

**Methods:**

We characterized the transcriptionally active network modules of interacting proteins in the vastus lateralis of COPD patients (n = 15, FEV_1_ 46 ± 12% pred, age 68 ± 7 years) and healthy sedentary controls (n = 12, age 65 ± 9  years), at rest and after an 8-week endurance training program. Network modules were functionally evaluated using experimental data derived from the same study groups.

**Results:**

At baseline, we identified four COPD specific network modules indicating abnormalities in creatinine metabolism, calcium homeostasis, oxidative stress and inflammatory responses, showing statistically significant associations with exercise capacity (VO_2_ peak, Watts peak, BODE index and blood lactate levels) (P < 0.05 each), but not with lung function (FEV_1_). Training-induced network modules displayed marked differences between COPD and controls. Healthy subjects specific training adaptations were significantly associated with cell bioenergetics (P < 0.05) which, in turn, showed strong relationships with training-induced plasma metabolomic changes; whereas, effects of training in COPD were constrained to muscle remodeling.

**Conclusion:**

In summary, altered muscle bioenergetics appears as the most striking finding, potentially driving other abnormal skeletal muscle responses.

*Trial registration* The study was based on a retrospectively registered trial (May 2017), ClinicalTrials.gov identifier: NCT03169270

**Electronic supplementary material:**

The online version of this article (10.1186/s12967-018-1405-y) contains supplementary material, which is available to authorized users.

## Background

Patients with chronic obstructive pulmonary disease (COPD) show marked individual variability of both clinical manifestations and disease progression with relevant implications on prognosis and management [1].

The 2017 GOLD update [2] recommends lung function measurements (FEV_1_) to assess COPD severity; whereas both symptoms intensity and history of COPD exacerbations are recommended indexes for the modulation of pharmacological therapy. However, these patients can also show systemic effects [3] and co-morbid conditions [4–6] that are independently associated with poor prognosis [1]. Enhanced knowledge of the underlying mechanisms of these two phenomena constitutes a key step toward a better understanding of COPD heterogeneity and its implications in patient management [7].

The current study focuses on the analysis of skeletal muscle dysfunction as a characteristic systemic effect of COPD, potentially modifiable with preventive interventions, i.e. exercise training [3, 4, 8–10]. Several studies addressed the question of training adaptation of COPD muscle, ranging from studies investigating expression of specific proteins [11–13] to modeling mitochondrial mechanisms [10] and systemic exploration of canonical pathways’ using gene expression [14, 15]. However, a comprehensive view of the disease mechanisms, highlighting potential biomarkers and pathway dynamics with functional implications is still missing. In our study, we applied a robust systems biology approach assuming that proteins associated to biological functions or diseases interact with each other conforming distinct neighborhoods, or network modules, in the human interactome [16, 17]. In other words, the network modules consist of clusters of active proteins (approximated in the study by transcriptionally active genes), showing high probability of functional interactions. We hypothesize that the identification, functional characterization and independent functional evaluation of such network modules can help to determine how their disturbance may lead to disease and how therapy may affect the molecular machinery [18].

**Fig. 1:**
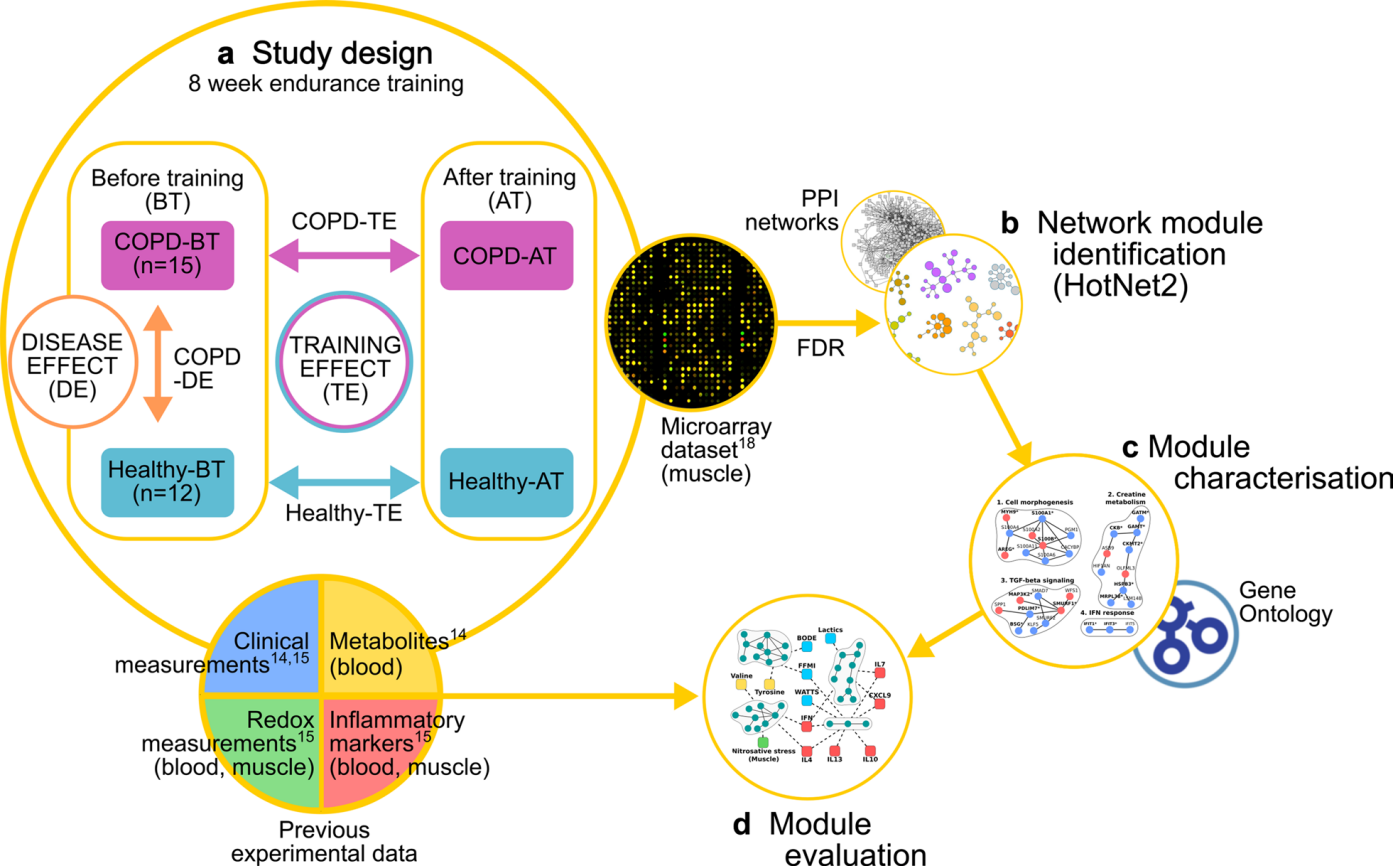
Schematic diagram of the workflow of the study. (a) Study design of the used datasets. COPD patients (n = 15) and healthy controls (n = 12) were studied before (BT) and after (AT) an 8-week endurance training program. Measurements of skeletal gene expression [15] were used for network modules identification. Differential conditions of COPD disease effects (COPD-DE) and training-induced effects in COPD (COPD-TE) and in healthy muscles (Healthy-TE) were analyzed in the study. (b) Network modules were identified for each differential condition with the HotNet2 algorithm [22], using the gene’s false discovery rate (FDR) adjusted differential expression P values and selected protein–protein interaction (PPI) networks [17, 23] as explained in details in Additional file 1: Section 1. Thereafter (c), each module was functionally characterized using gene ontology (GO) term enrichment analysis. (d) Correlation of network modules with independent multilevel measurements was analyzed for evaluation purposes. Specifically, independent measurements were sampled both pre- and post-training and consisted of physiological parameters measured with a constant-work rate exercise at 75% of pre-training maximum peak exercise, inflammatory and redox biomarkers measured in plasma and in skeletal muscle [20], as well as plasma metabolomics measured at rest and after exercise [19]

To further explore the underlying mechanisms of skeletal muscle dysfunction, we compared healthy persons and COPD patients before and after exercise training. In the pre-training analysis (Fig. 1), we described transcriptionally active network modules that are specific to the skeletal muscle of COPD patients. Likewise, in the assessment of adaptive mechanisms of endurance training, we compared the differences between COPD and healthy muscle adaptation. Functional implications were initially explored through the analysis of network module associated pathways and representative differentially expressed genes. In a subsequent step, we evaluated the functional interpretation of the network modules, and relevant genes, with previous experimental data obtained in the same study groups [19, 20].

To the best of our knowledge the current research provides an innovative approach by retrieving disease specific pathway mechanisms and performing an integrative analysis of the relationships of transcriptomics with metabolic, redox, inflammatory and clinical measurements to investigate COPD muscle dysfunction and training-induced adaptive changes in these patients. We believe that the study sheds novel light on underlying mechanisms of the disease with potential implications for the design of innovative preventive strategies.

## Methods

### Study dataset

The current study is based on a dataset of microarray gene expression measurements (Human U133 Plus2 Gene Chips) performed on open biopsies from the limb muscle vastus lateralis, reported in [15]. In all participants, these were obtained at rest, before and after an 8-week high intensity endurance training program (Fig. 1a). The study groups (Table 1) included fifteen COPD patients and twelve healthy but sedentary age-matched controls. The training program is explained in details in Additional file 1: Section 1 and in the related studies [15, 19, 20].

**Table 1:** Characteristics of the study groups

	Healthy	COPD
Sex (M/F)	10/2	15/0
Age, years	65 ± 9	69 ± 7
FFMI, kg/m^2^	21 ± 2	19 ± 3
FEV_1_, L (mean % pred)	3.46 ± 0.69 (107)	1.34 ± 0.37 (46)*
FEV_1_/FVC	0.75 ± 0.04	0.43 ± 0.08*
VO_2_ peak, L/min (mean VO_2_ peak/kg)	1.70 ± 0.5 (22)	0.91 ± 0.3 (14)*
[La]a peak, mEq/L	10.60 ± 2.7	6.8 ± 2.3*
VO_2_ peak training diff (post–pre), L/min	0.25 ± 0.11^†^	0.14 ± 0.18^†^
[La]a training diff (post–pre), mEq/L	− 4.60 ± 0.6^†^	− 1.5 ± 2^†^

Results are expressed as mean ± SD

In the post-training study, lactate measurements during constant-work rate exercise were done at the same workload and duration than the pre-training exercise protocol

*FFMI* fat free mass index, *FEV*_*1*_ forced expiratory volume in the first second, *FEV1/FVC* FEV_1_ to forced vital capacity ratio, *VO*_*2*_ *peak* peak oxygen uptake difference post minus pre-training, *[La]a* arterial lactate concentration difference

Unpaired t test was used to compare controls and COPD, * P < 0.05. Paired t test was used to compare post-training and baseline time points in both healthy controls and COPD patients, ^†^P < 0.05. Low FFMI was defined as < 17.05 kg/m^2^ for men [21]. It is of note that three COPD patients were discarded from the analysis because they did not pass the Agilent analysis

### Analysis strategy

Briefly, network modules were identified for each differential condition with the HotNet2 algorithm [22] (Fig. 1b), using the genes’ adjusted differential expression profile and selected protein–protein interaction (PPI) networks [17, 23]. Thereafter (Fig. 1c), each module was functionally characterized using gene ontology (GO) [24] term enrichment analysis and literature mining. Finally, (Fig. 1d), the validity of the matched module functions was evaluated using previous experimental data (Tables 1, 2).

**Table 2:** Summary of experimental data obtained from the same study groups

Measurements	COPD versus health Summary of results
Plasma metabolomics [19]	The two groups showed differences in metabolomic profiles at rest (P < 0.05). Levels of valine, alanine and isoleucine were associated with FFMI (P < 0.01 each)
Plasma metabolomics training diff [19]	In Healthy, training generated marked changes in amino acids, creatine, succinate, pyruvate, glucose and lactate (P < 0.05 each). But, COPD patients only showed lactate decrease (P < 0.05)
Inflammatory cytokines [20]	COPD patients showed high levels of circulating cytokines (P < 0.05), not seen in healthy
Inflammatory cytokines training diff [20]	No training-induced changes were observed in circulating cytokines levels
Redox status [20]	COPD patients showed blood and muscle oxidative stress at baseline. Muscle and blood protein carbonylation levels were correlated (P < 0.05)
Redox status training diff [20]	In COPD patients, protein nitration levels decreased after training

Summary description of the results of previous experimental measurements on plasma metabolomics [19], as well as on both muscle and blood inflammatory cytokines and redox status [20], carried out at rest before training and after endurance training. The term training diff refers to training-induced adaptive changes. For comprehensive list of measured variables see Additional file 2: Tables S2, S7 for the differentials

### Statistical analysis

#### Differential gene expression

To evaluate the baseline (pre-training) effects, we computed the differential gene expression between COPD and healthy individuals, referred as COPD disease effects (COPD-DE) (Fig. 1a). To evaluate the training induced changes in the molecular mechanisms (training effects, TE), we investigated the post and pre-training differential gene expression in COPD (COPD-TE) and healthy (Healthy-TE) separately (Fig. 1a). The non-parametric rank product method [25] was used to compute the significance and false discovery rate (FDR) of differential gene expression, due to its reliable and consistent performance with noisy, low sample size measurements.

#### Network module identification

For each condition, we used the HotNet2 algorithm [22] to identify network modules (Fig. 1c), taking into account: (i) the FDR of differential gene expression and, (ii) publicly-available high quality protein–protein interaction (PPI) networks [17, 23] (see Fig. 1b). A statistical test included in the HotNet2 algorithm was used to determine the significance of the number and size of the network modules. The HotNet2 algorithm was selected due to its specific feature of the use of a heat diffusion/random walk model to simulate the spread of influence of protein activity to their physical interaction partners. This feature makes this approach less reliant on the significance test and enables the identification of key proteins with less significant changes but with high biological meaning (i.e. due to topology: hub proteins, high betweenness centrality proteins, etc.). For more details see extended methods in Additional file 1: Section 1.

#### Functional characterization

We conducted Gene Set Analysis to investigate the enrichment of GO terms in modules (Fig. 1c) using the clusterProfiler R library [26]. Network modules were considered functionally significant if it had at least one associated GO term that: (i) had Benjamin–Hochberg corrected P value < 0.05; and, (ii) were related to at least two module genes.

#### Evaluation of the module functions with experimental data

To evaluate the identified functions, modules were compared with experimental data obtained in the same study group (Fig. 1a, Tables 1, 2), firstly the transcriptional activity of the network modules were summarized using their first three principal components, i.e. their first three eigengenes [27, 28], which on average explained 83% of the modules’ overall variability. Then, associations of the principal components with the previous experimental data [19, 20] were identified using non-parametrical Kendall correlation and selecting those associations with absolute value of *rho* (*|R|*) ≥ 0.4 and P value (P) < 0.05. Significant differentially expressed genes within functionally significant network modules were also considered for comparison with previous experimental data.

## Results

### Study workflow

In the pre-training analysis, we describe transcriptionally active network modules that are specific to COPD patients (Fig. 1a–c). Likewise, in the analysis of the training-induced effects, we separately analyze network modules that changed in response to training in COPD and healthy and compare the differences between them. Functional implications of the network modules were initially determined through the analysis of pathways associated to module genes and then specific mechanisms were deduced from the gene functions and interactions. In a subsequent step, the network modules and representative genes of specific pathways are compared with previous experimental data obtained in the two study groups both showing clear training-induced physiological responses, as described in Fig. 1d and in Tables 1 and 2.

### Alterations in skeletal muscle of COPD patients at rest

**Fig. 2:**
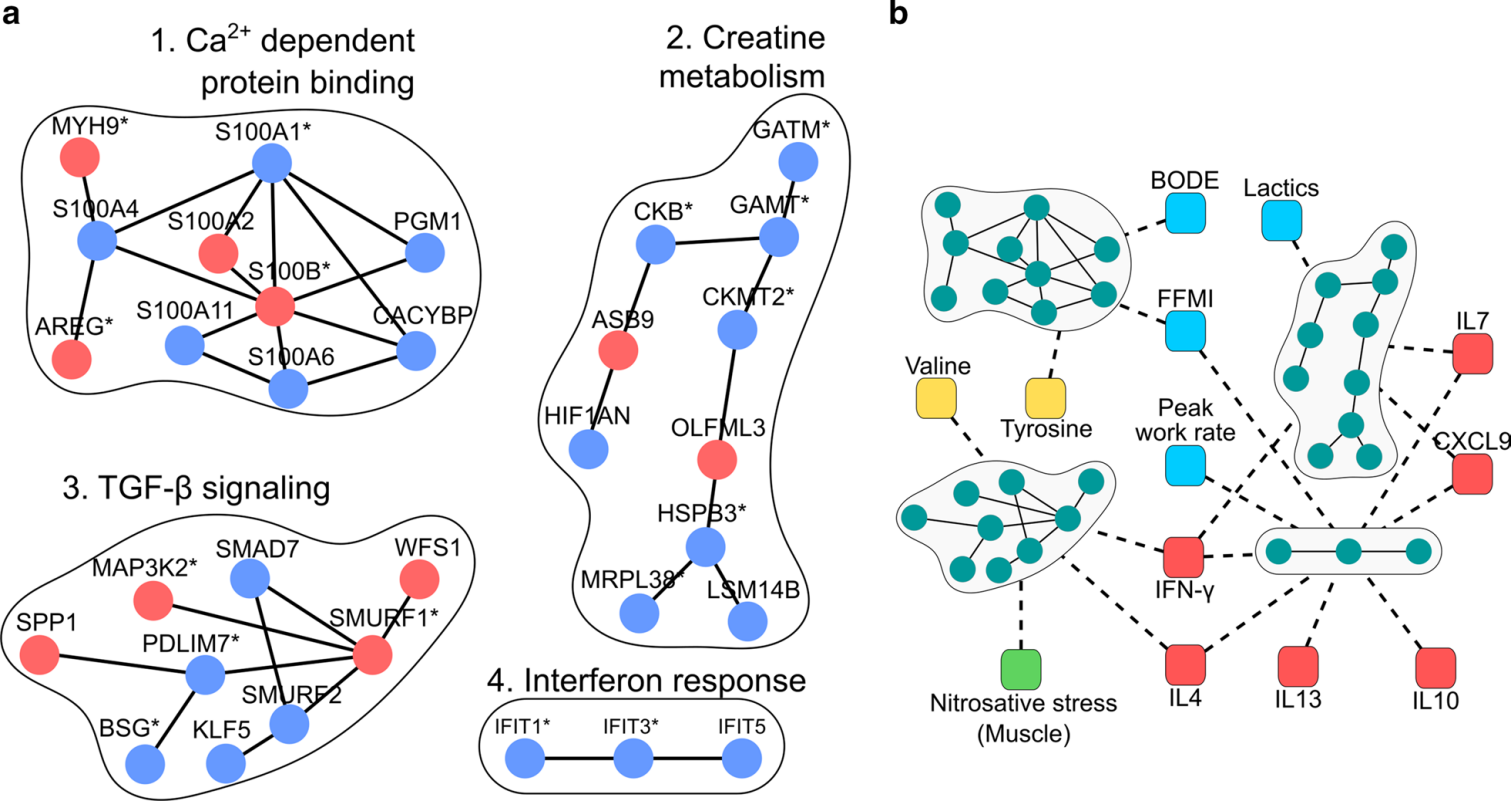
Disease effects (COPD-DE) network modules. **a** The four network modules associated to COPD disease effects and their composing genes. Genes are colored according to their differential regulation, namely: up regulation—red nodes; and down regulation—blue nodes. Significantly differentially expressed genes are indicated by * (FDR ≤ 0.05) (for detailed information see Additional file 2: Table S6). **b** The significant correlations of independent measurements with any of the network modules’ first three principal components. Blue squares depict exercise related independent variables [19]; red squares show cytokines measured in blood [20]; yellow squares correspond to amino acids measured in serum [19]; and, green squares represents redox biomarkers [20]

The pre-training study identified four significant COPD specific network modules, that were functionally characterized, on the basis of significantly enriched GO terms in the modules (see Additional file 2: Table S5), as: creatine metabolism, Ca^2+^ dependent binding, TGF-β signaling and Interferon response (Fig. 2a).

Defective skeletal muscle energy metabolism in COPD was indicated by the creatine metabolism module. The module presented four out of the nine genes of the creatine metabolism pathway significantly down-regulated, two related to creatine synthesis (GAMT, GATM) and two creatine kinase (CK) genes (CKB, CKMT2). Overall, down-regulation of creatine metabolism suggests impairment of muscle energy production, which is consistent with studies showing low baseline creatine kinase and ATP concentrations [29, 30]; and low post-exercise recovery rate in COPD skeletal muscle [31–33].

It is of note that impaired creatine metabolism would primarily affect work performance and Ca^2+^ homeostasis, especially in the presence of oxidative stress [34, 35]. In line with this, we found clustering of S100 family calcium-dependent protein binding genes in the Ca^2+^ dependent protein binding module. The potential deleterious effect of the module is well represented by the down-regulation of S100A1 gene, which could lead to abnormal sarcoplasmic reticulum Ca^2+^ content and fluxes, deteriorating muscle contractility and work performance [36, 37]. Furthermore, several module genes (S100B, S100A4, S100A6, MYH9) are related to cell morphogenic processes.

The TGF-β signaling module displayed an interplay of genes related to muscle remodeling (SMURF1, SMURF2, SMAD7) and cellular stress response (MAP3K2, SPP1). Abnormal TGF-β signaling was suggested by up-regulation of its inhibitor SMURF1 and further strengthened by the observed down-regulation of SMURF1’s binding competitor (PDLIM7) [38] potentially leading to increased protein degradation by ubiquitination [39]. The associated gene functions suggest an interplay between TGF-β signaling and oxidative stress, which has been reported in the literature highlighting the specific role of SMURF1 in these processes [40, 41]. Furthermore, overexpression of SMURF1 may attenuate IFN-γ-mediated immune responses of the Jak-STAT pathway, by inhibiting STAT1 [42, 43], positive regulator of IFIT gene expression [44], which could explain systematic down-regulation of these genes in the interferon response module.

### Evaluation of alterations in COPD patients at rest

In order to evaluate the functions of the COPD specific network modules, their association with previous experimental data was analyzed (see Fig. 2b and for details Additional file 2: Table S8).

The Creatine metabolism module showed statistically significant associations with systemic inflammatory markers, namely IFN-γ (|R| = 0.42, P = 0.041), IL7 (|R| = 0.5, P = 0.016) and CXCL9 (|R| = 0.58, P = 0.003) as well as with pre-training blood lactate levels at a constant-work rate exercise at 75% VO_2_ peak (|R| = 0.49, P = 0.013) suggesting relationships between altered cell bioenergetics and abnormal inflammatory processes.

**Fig. 3:**
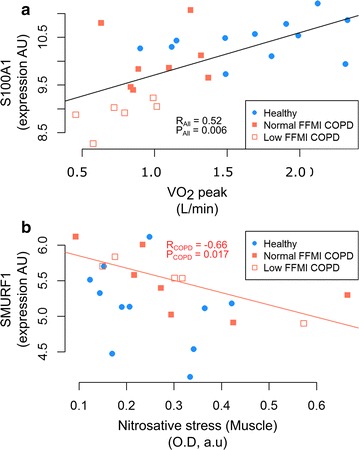
Relationships between genes from COPD specific modules (disease effects) and previous experimental data. **a** The relationships between S100A1, from the Ca^2+^ dependent binding module, and VO_2_max. The two groups, healthy subjects (blue circles) and COPD patients (low and normal FFMI, empty and filled squares, respectively) fell on the same regression line (R = 0.52, P = 0.006, FDR = 0.026). **b** The relationships between SMURF1 from the TGF-β signaling module and skeletal muscle nitrosative stress. A statistical significant correlation was seen in the COPD group, both normal and low FFMI (R = − 0.67, P = 0.018 and FDR = 0.07), but not in healthy subjects (R = − 0.2, P = 0.55)

The association of the Ca^2+^ dependent protein binding module with muscle mass (FFMI) (|R| = 0.45, P = 0.026) and with exercise capacity, expressed by the composite BODE index [45] (|R| = 0.47, P = 0.033) confirms the physiological impact of defective Ca^2+^ homeostasis. Such an association at module level is further strengthened by the correlations of the S100A1 gene expression with both VO_2_ peak (R = 0.52, P = 0.006) (Fig. 3a) and peak work rate (Watts peak) (R = 0.53, P = 0.005).

Consistent with the functional analysis, TGF-β signaling module showed significant correlations with increased skeletal muscle nitrosative stress in COPD patients (|R| = 0.49, P = 0.031), as well as with abnormally low levels of blood valine amino acids (|R| = 0.41, P = 0.047). Likewise, blood cytokines IFN (|R| = 0.44, P = 0.03) and IL4 (|R| = 0.47, P = 0.024) also showed significant associations with the module. At gene level, statistically significant negative correlations were observed between SMURF1 and nitrosative stress levels in skeletal muscle of COPD patients (R = − 0.66, P = 0.017), not seen in healthy subjects (Fig. 3b).

As expected, interferon response module showed significant correlations with IFN (|R| = 0.63, P = 0.015) and several other cytokines, which presented elevated blood levels in COPD patients (Table 2). Furthermore, the module also presented significant relationships with FFMI (|R| = 0.47, P = 0.019) and peak work rate (|R| = 0.40, P = 0.047) in COPD patients.

### Inefficient training-induced responses in COPD patients

**Fig. 4:**
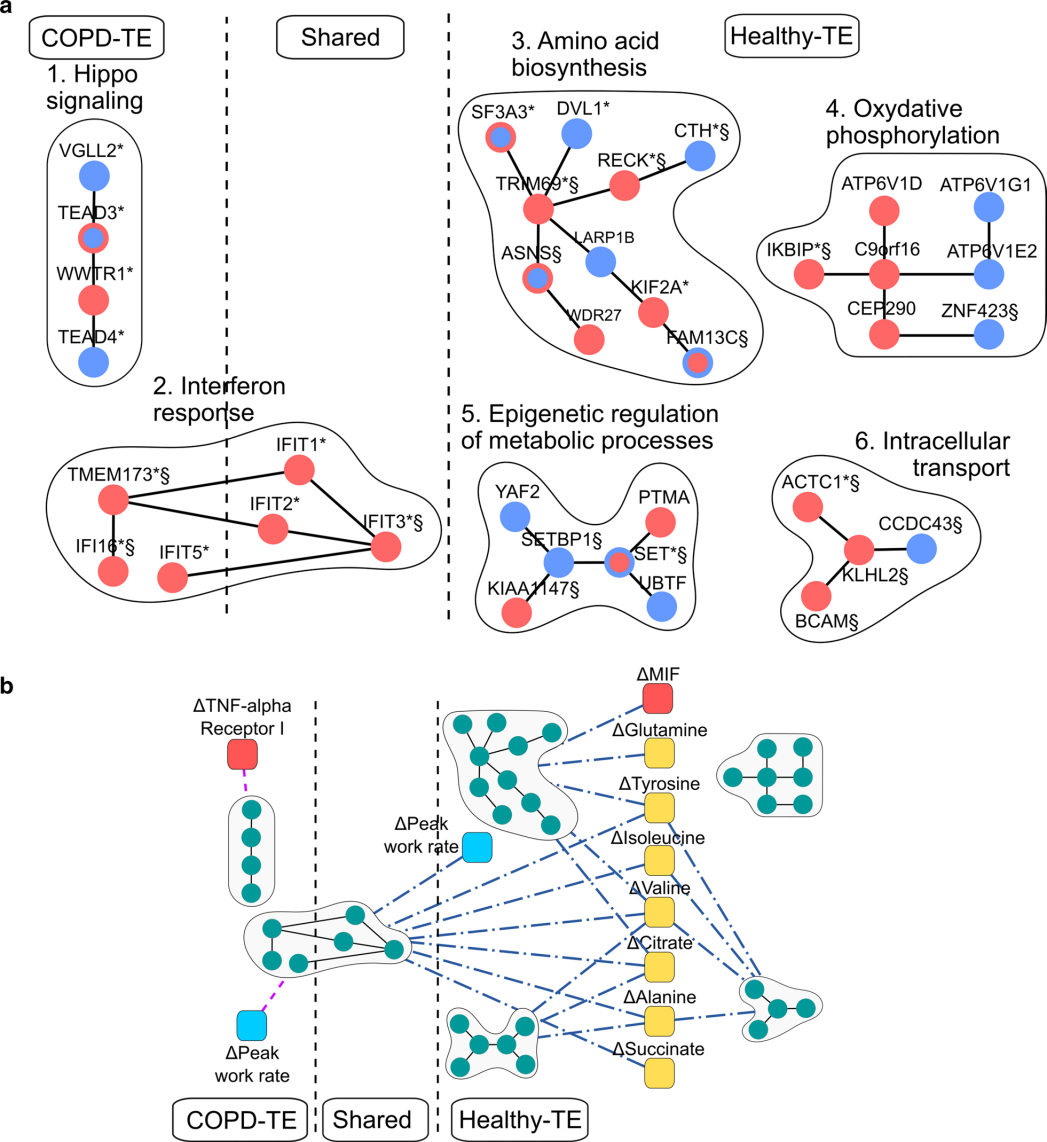
Training effects (TE) network modules. **a** Active network modules identified in case of COPD-TE, Healthy-TE and in both (shared). Genes are colored according to their differential regulation in COPD-TE (inner color of the nodes) and in Healthy-TE (border color of the nodes): up regulation with training (red circles), down regulation with training (blue circles). Modules are named after significantly enriched GO terms. Training differential expression significance is signed by * for COPD-TE, and ^§^ for Healthy-TE (FDR < 0.05) (for detailed information see Additional file 2: Table S6). **b** The significant correlations of the independent measurements with any of the significantly-changed training modules’ first three principal components in COPD, depicted as purple dashed lines, and in healthy subjects, depicted as blue dotted-dashed lines. Blue squares depict exercise related independent variables; red squares show cytokines measured in blood; and yellow squares correspond to amino acids measured in serum

In the analysis of the training-induced effects (TE), we identified and evaluated network modules separately for COPD patients (COPD-TE) and for healthy sedentary subjects (Healthy-TE). The research identified a total of six functionally enriched network modules (Fig. 4a).

It is of note that Hippo signaling was the only COPD-TE specific module; whereas, some genes of the Interferon response were observed in both COPD-TE and Healthy-TE. Likewise, Oxidative phosphorylation, Amino acid biosynthesis, Epigenetic regulation of metabolic processes and Intracellular transport functional modules were only observed in Healthy-TE and were named after significantly enriched GO terms in the modules.

In COPD-TE, the Hippo pathway module suggests abnormal training-induced activation of skeletal muscle remodeling, as reported in detail in the extended results section in Additional file 1: Section 1.

Endurance training induced inflammatory responses in skeletal muscle, as indicated by the Interferon response module that showed a consistent increase in gene expression levels in both COPD-TE and Healthy-TE. The module could signal the local inflammatory response to muscle damage caused by exercise, which reportedly coincides with muscle repair, regeneration, and growth [46].

It is of note that the four Healthy-TE network modules indicated strong associations of training responses with bioenergetics changes and their joint regulation with other molecular functions (see extended results in Additional file 1: Section 2).

### Evaluation of training-induced responses in COPD patients

**Fig. 5:**
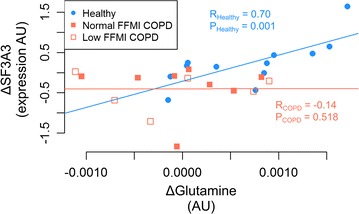
Relationships between genes from Healthy-TE specific modules and previous experimental plasma metabolomics data. The figure depicts the relationships between training-induced changes in both SF3A3, from the Amino acid biosynthesis module, and glutamine. A strong correlation was seen in healthy subjects (blue circles) (R = 0.70, P = 0.001), but not in COPD patients (low and normal FFMI, empty and filled red squares, respectively) (R = − 0.14, P = 0.518)

The analysis of associations between TE network modules and previous experimental data was carried out in COPD-TE and Healthy-TE separately, as displayed in Fig. 4b (for details see Additional file 2: Table S8). We observed a significant association between training-induced increase in peak work rate (Watts) and the interferon response module in the two groups (|R|_COPD_ = 0.48, P_COPD_ = 0.019; |R|_Healthy_ = 0.53, P_Healthy_ = 0.018), suggesting training-induced increase of inflammatory responses both in healthy subjects and in COPD patients. However, the most relevant findings were the strong relationships between Healthy-TE network modules associated to different aspects of muscle bioenergetics and metabolomics training-induced changes, not seen in COPD patients. Likewise, statistically significant associations were observed between training-induced transcriptional changes at gene level and plasma metabolomics responses in healthy subjects, but not in COPD patients, as shown in Fig. 5 wherein the relationships between training-induced changes the splicing factor SF3A3 (∆SF3A3) and ∆glutamine are depicted for healthy subjects (R_Healthy_ = 0.7, P_Healthy_ = 0.001) and for COPD patients (R_COPD_ = − 0.14, P_COPD_ = 0.518).

## Discussion

The approach adopted in the current study contributed to uncover novel interactions among biological pathways of skeletal muscle dysfunction in COPD patients, as well as suggest biomarkers, while reinforcing previous results on the mechanisms related to the disease. The applied methodological framework also shows high potential to explore relations between clinical and omics platforms, facilitating interpretation of biological measurements.

The four network modules identified in the pre-training analysis (Fig. 2a) correspond to COPD specific mechanisms related to abnormal energy production and contractility, as well as to alterations in both inflammatory and oxidative stress pathways. Moreover, they showed significant associations with previous measurements carried out in the same study group (Figs. 2b, 3). To be noted that lung function (FEV_1_) only presented a weak negative relationship with the interferon module that did not meet the inclusion criteria of the analysis. In contrast, several COPD specific modules, and genes (Fig. 2b) consistently showed associations with different indices reflecting exercise capacity, namely: BODE, VO_2_ peak, Watts peak and lactate levels.

The two study groups showed significant physiological training effects as displayed in Table 1. The differences in the training-induced responses between COPD and healthy (Fig. 4a) further contributed to shed novel light on the underlying mechanisms of skeletal muscle dysfunction in these patients. The most striking finding was that the physiological bioenergetics responses, strongly correlated with plasma metabolomics (Fig. 4b), were not observed in the patients. Instead, in the COPD group, the training-induced changes were mostly related with skeletal muscle remodeling (Hippo signaling pathway), without significant adaptive changes in oxidative phosphorylation and related bioenergetics pathways. It is of note that in a post hoc analysis, we explored the impact of FFMI on the modules, which consistently indicated that training adaptation seen in COPD patients with normal FFMI were more similar to the ones of healthy subjects than those observed in COPD patients with low FFMI (see Additional file 1: Section 2). Regarding the training-induced inflammatory responses, the healthy and COPD groups only shared part of the genes of the network module that indicates increased inflammatory changes induced by training in COPD. It is of note that significant associations of peak work rate with the inflammatory network modules were observed in the disease effects (Fig. 2a, b) and in the training-induced effects (Fig. 4a, b).

As acknowledged below, the current study cannot inform on causality and temporal sequence of the skeletal muscle abnormalities observed in the COPD group. The marked differences between COPD patients and healthy subjects regarding training adaptations of skeletal muscle bioenergetics (Fig. 4a) seem to suggest that the abnormal energy production, already depicted in the pre-training analysis (Fig. 2a), is the most visible and likely the primary phenomenon of skeletal muscle dysfunction in COPD. It is of note that a recent report using data from the same study group [10], but focusing on the analysis of gene regulatory networks, highlighted the existence of significant COPD abnormalities at mitochondrial level with impact on skeletal muscle inflammatory responses, and explored potential therapeutic strategies.

Abnormal bioenergetics may likely trigger changes in skeletal muscle Ca^2+^ homeostasis, which ultimately may lead to impairment of the contractile mechanisms and alterations in muscle morphogenesis, as suggested by the Ca^2+^ dependent protein binding module (Fig. 2a) and the Hippo signaling pathway module (Fig. 4a). These mechanisms might be related to generation of abnormal muscle fiber type distribution with increased glycolytic (Type II and IIX) to oxidative (Type I) fiber ratio in these patients [47, 48]. In the study, physiological inflammatory response pathways at baseline showed to be inhibited, potentially by SMURF1, and most likely be modulated by oxidative stress, which might indicate counter-regulatory processes related with low-grade systemic inflammation. The partly abnormal training-induced inflammatory responses observed in the study might also constitute a secondary phenomenon modulated by nitroso-redox disequilibrium reported in these patients [3, 8, 20].

The network biology techniques used in the current study to identify and characterize skeletal muscle network modules are gaining increasing attention in the biomedical research field due to their ability to highlight complex cellular disease mechanisms [49–51]. An added potential of PPI based methods is the constraint that the interaction network represents, whose topology already encodes basic biological functions [16, 52] and provides high performance in predicting biologically meaningful pathways [53]. Additionally, the model used in HotNet2, simulating the spread of influence of protein activity, enables the identification of key proteins with less significant changes but with high biological meaning due to surrounding expression patterns as well as due to topology (e.g. hub proteins, proteins with high betweenness centrality, etc.), which complement standard differential expression measures with deeper biological insights. We believe that the approach adopted in the current study facilitates a comprehensive analysis and understanding of complex cellular mechanisms overcoming limitations of traditional research only addressing analysis of target biological pathways. Furthermore, the applied methodology has high potential for creating a standardized analysis pipeline for the integrative analysis of multi-level data.

### Study limitations

We acknowledge, however, that further longitudinal studies are needed to support the above statements, as well as to properly clarify the relationships between skeletal muscle dysfunction and pulmonary impairment provoked by the disease. We also acknowledge that the microarray dataset used in the study is lacking standard qPCR validation of specific biomarkers, which we aimed to overcome by showing the high concordance of specific markers with qPCR validation of two earlier studies on skeletal muscle of patients with COPD (Additional file 2: Table S9). However, we believe that given our system-based approach, the validation of a few genes is less relevant compared to the functional evaluation of the modules with independent measurements that was conducted in the study. Furthermore, the completeness and/or bias of the publicly available PPI networks [17, 23] are intrinsic limitations of the methodological approach which, additionally, does not provide information on causality. The rather small sample size constituted a problem such that a type II error limiting our interpretations of the results cannot be excluded. Furthermore, the limitation of sample size has been addressed using robust statistical approaches at each step of the analysis. In particular, when choosing the HotNet2 algorithm and its application, as explained in detail in the extended methods and, in general, when considering protein–protein interactions (PPI) network based methods, which offer a more robust performance in small sample size environments [54] compared to other systems medicine approaches [15]. Moreover, the identification of both statistically and biologically significant relationships of the resulting functional modules (and genes) with previous experimental multilevel data obtained in the same study groups [19, 20] provided additional robustness to the evaluation and functional characterization of the core findings of the study. Summing up, different factors emerging from the study design, such as sample size, noisy clinical environment and factors originating from the modeling technique in use, such as (i) current constraints of available PPI networks, (ii) modeling proteins levels with gene expression, (iii) relying on arbitrary significance thresholds, and (iv) comparing of measurements of different body compartments (blood, muscle) may lead to confounding results, which prompts for future validation of the study. The consistency of the results, however highlights the potential of biological modeling as a preliminary step for future discoveries. The above mentioned factors may also explain that the study did not identify specific pathways that are known to play a significant role in skeletal muscle dysfunction in COPD, such as the FoxO signaling pathway [3, 14, 55].

We acknowledge that differences in training intensity between healthy subjects and COPD patients (Table 1) should be considered in the interpretation of the results. However, the findings of the study are supported by the following factors: (i) pre-training COPD specific findings; and, (ii) qualitative nature of the training-induced differences between healthy and COPD unlikely explained only by differences in training intensity. A final methodological consideration is that the COPD group includes only males, which constitute an over-representation of this gender (Table 1), as compared to current COPD prevalence in men. However, no reports on gender specificity of the findings have been found neither in the literature nor in our dataset (Additional file 1: Figure S5).

### Future work

We believe that the current study significantly contributed to enhance our understanding of skeletal muscle dysfunction in patients with COPD. Further research addressing the molecular mechanisms of impaired muscle energy production in these patients should shed light on remaining challenges such as, causality, lung-muscle interactions and design of cost-effective strategies aiming at preventing non-pulmonary effects in COPD patients. The central role of impaired bioenergetics seems to endorse that promotion of daily physical activity at early disease stages may have a role preventing skeletal muscle dysfunction in these patients. We believe that future longitudinal studies using the current methodological approach will generate further evidence supporting our interpretations of the current study findings.

A better knowledge on underlying mechanisms of non-pulmonary effects of COPD should necessarily lead to enhanced patient risk assessment and better health service selection. Moreover, continuous progresses in our understanding of mechanisms of COPD heterogeneity might prompt the need for revisiting the taxonomies of obstructive airways diseases.

## Conclusions

The research provides a comprehensive view of the core mechanisms involved in skeletal muscle dysfunction as a systemic effect of COPD. The results indicate that COPD patients show impaired training-induced responses in skeletal muscle bioenergetics, with abnormal inflammatory changes and altered tissue remodeling, as compared to healthy sedentary subjects. The current network medicine approach shows high potential for future longitudinal analyses exploring preventive strategies addressing non-pulmonary effects of COPD.

## Additional files

**Additional file 1: Section 1.** Expanded methods: TRAINING program, HotNet2 and parameters, Interactome construction. **Section** **2.** Expanded results: Training induced adaptation, Impact of FFMI on disease effect modules, Impact of FFMI on training effect modules. Training effect differences between COPD and healthy. Gender effect on the transcriptomics profile. **Figure S1.** Overlap between nodes and edges in the interaction networks. **Figure S2.** Disease effect modules of the COPD subgroups. **Figure S3.** Training effect modules of the COPD subgroups. **Figure S4.** Difference between COPD and healthy training effects. **Figure S5.** Gender effect on the transcriptomics profile.

**Additional file 2: Table S1.** Number of differentially expressed genes in the different groups and conditions. **Table S2.** Previous measurements: List of variables measured. **Table S3.** Network modules: HotNet2 results. **Table S4.** Network modules: HotNet2 consensus. **Table S5.** Network modules: Functional characterization. **Table S6.** Network modules: Gene differential expression. **Table S7.** Previous measurements: Differentials. **Table S8.** Previous measurements: Association with network modules. **Table S9.** Comparison of microarray results with qPCR validation of external datasets.

## Abbreviations

COPD: chronic obstructive pulmonary disease
COPD-DE: COPD disease effects
COPD-TE: COPD training effects
Healthy-TE: healthy training effects
COPDN: normal FFMI COPD
COPDL: low FFMI COPD
FDR: false discovery rate
PPI: protein–protein interaction
GO: gene ontology
FFMI: fat free mass index
FEV1: forced expiratory volume in the first second
FEV1/FVC: FEV1 to forced vital capacity ratio
VO_2_ peak: peak oxygen uptake difference post minus pre-training
[La]a: arterial lactate concentration difference
WATTS: peak work rate
