# Supplementary material for: Network modules uncover mechanisms of skeletal muscle dysfunction in COPD patients
Source: J Transl Med. 2018 Feb 20;16:34. doi: 10.1186/s12967-018-1405-y (PMC5819708; doi:10.1186/s12967-018-1405-y)
Supplement: Supplementary file 1 — Additional file 1: Section 1. Expanded methods: TRAINING program, HotNet2 and parameters, Interactome construction. Section 2. Expanded results: Training induced adaptation, Impact of FFMI on disease effect modules, Impact of FFMI on training effect modules. Training effect differences between COPD and healthy. Gender effect on the transcriptomics profile. Figure S1. Overlap between nodes and edges in the interaction networks. Figure S2. Disease effect modules of the COPD subgroups. Figure S3. Training effect modules of the COPD subgroups. Figure S4. Difference between COPD and healthy training effects. Figure S5. Gender effect on the transcriptomics profile. [file 12967_2018_1405_MOESM1_ESM.docx]

**Network modules uncover mechanisms of skeletal muscle dysfunction in COPD patients**

*Ákos Tényi, Isaac Cano, Francesco Marabita, Narsis Kiani,Susana Kalko, Esther Barreiro, Pedro de Atauri, Marta Cascante and David Gomez-Cabrero, Josep Roca*

**Additional file 1**

Outline

[SECTION 1 – EXPANDED METHODS 3](#_Toc475698711)

[Training program 3](#_Toc475698712)

[Network modules 3](#_Toc475698713)

[HotNet2 4](#_Toc475698714)

[HotNet2 parameters. 4](#_Toc475698715)

[Identification of consensus network modules. 5](#_Toc475698716)

[Comparison of HotNet2 to other algorithms. 5](#_Toc475698717)

[Interactome construction 5](#_Toc475698718)

[SECTION 2 – EXPANDED RESULTS 7](#_Toc475698719)

[Training-induced adaptations 7](#_Toc475698720)

[Impact of FFMI on disease effect (DE) modules 7](#_Toc475698721)

[Impact of FFMI on training effect (TE) modules 8](#_Toc475698722)

Training effects [differences between COPD and healthy 8](#_Toc475698723)

[Gender effect on the transcriptomics profile 8](#_Toc475698724)

[ONLINE SUPPLEMENT REFERENCES 10](#_Toc475698725)

[SUPPLEMENTARY FIGURES 13](#_Toc475698726)

**[Figure S1.](#_Toc475698727)** [Overlap between nodes and edges in the interaction networks. 13](#_Toc475698727)

[**Figure S2.** Disease effect modules of the COPD subgroups. 13](#_Toc475698728)

[**Figure S3.** Training effect modules of the COPD subgroups. 14](#_Toc475698729)

[**Figure S4.** Difference between COPD and healthy training effects. 14](#_Toc475698730)

**[Figure S5.](#_Toc475698731)** [Gender effect on the transcriptomics profile. 15](#_Toc475698731)

SUPPLEMENTARY TABLES (see separate Excel file)

**Table S1.** Number of differentially expressed genes in the different groups and conditions.

**Table S2.** Previous measurements: List of variables measured

**Table S3.** Network modules: HotNet2 results

**Table S4.** Network modules: HotNet2 consensus

**Table S5.** Network modules: Functional characterization

**Table S6.** Network modules: Gene differential expression

**Table S7.** Previous measurements: Differentials

**Table S8.** Previous measurements: Association with network modules

# SECTION 1 – EXPANDED METHODS

## Training program

COPD patients and controls exercised on a cycle ergometer (Jaeger ER 550; Wüerzburg, Germany) five days per week for eight weeks following the recommendations of the American Thoracic Society guidelines^2,3^. The training sessions with cycle ergometry, of 1 h duration, consisted of: (1) warm-up: 5 min of cycling at 30% Watts (W) peak; (2) exercise: 40 min cycling of interval training combining 2 min at high work rate, 70–100% Wpeak, with 3 min at 40–50% Wpeak; followed by (3) cold-down consisting of 5 min cycling at 30% Wpeak.

## Network modules

In complex diseases, proteins do not act in isolation, rather, they interact together in pathways and modules to perform the designated function^4^. Computational techniques aiming at identifying such modules in protein-protein interaction (PPI) networks are gaining increasing attention in the biomedical research field due to their ability to highlight complex cellular mechanisms^5–7^. Moreover, in low sample size they show more robust performance compared to other methods, such as network inference^8^. An exciting new technique in this field aims to simulate the spread of influence of protein activity – often substituted by gene expression data – to their physical interaction partners, aiming at retrieving biologically more meaningful modules^6^. In this study, we aim to explore the underlying mechanisms of COPD muscle dysfunction using an algorithm that implements this technique.

## HotNet2

In the study HotNet2 algorithm^9^ was used to identify transcriptionally active modules. HotNet2 has been mostly applied in cancer mutational profile characterization^9–11^, however it was also proposed as a general framework, applicable for transcriptomics data analysis^12^. The algorithm is based on a modified heat diffusion model, where proteins function as heat sources and physical interactions amongst them are links where heat can diffuse, i.e. simulating the spread of the influence of each protein to its interactors. To define the plane of interactions, protein-protein interaction (PPI) networks were used (described below). To define the “heat” of the proteins, heat scores were computed as$\sigma\left( g \right)=-{log}_{10} q_{g}$, where *q_g_* is the gene *g*‘s false discovery rate (FDR). Identification of network modules were done by extracting strongly connected components in the network, where proteins diffuse >*δ* “heat” to each other, i.e. hot modules. Finally, HotNet2 employs a statistical test to determine the significance of the number and size of the network modules identified in the previous step.

## HotNet2 parameters.

HotNet2 has two parameters: β and δ. β expresses the rate of heat diffusion from a protein to its neighbors and to the rest of the network and is selected for a given protein-protein interaction network, independently of any heat scores. The β parameter was selected by identifying network modules in the HI network (see below) using several βs (β = {0.40, 0.45, 0.50}) identified using the guidelines of the original HotNet2 publication (E6) and selecting the β with the most significant modules identified (β = 0.45). This value was applied on all the three PPI networks. From the several δ output generated by the HotNet2, the δ with the lowest identified p-value (obtained with 100 permutations) was selected. The selected δ are shown in **Additional file 2:** **Table S3** in the separate supplementary tables file.

## Identification of consensus network modules.

To achieve more robust results and to minimize the inherent bias introduced by the PPI networks, network modules were identified with HotNet2 using three different PPI networks and then their consensus network modules were computed^9^. Consensus modules are built by first identifying the core network module, which was reported in all the three PPI networks. Then proteins reported in two and then one PPI networks are iteratively added to this core network. In the analysis, only the identified consensus networks were reported. For the list of identified modules, see **Additional file 2:**  **Table S4** and **Table S6**.

## Comparison of HotNet2 to other algorithms.

Other alternative approaches for gene regulatory network reconstruction were considered^13^, however our initial attempts showed over-sensitivity of the results to the different sample size of our study groups, which HotNet2 together with a robust differential gene expression test (as explained in the main text) was able to avoid.

## Interactome construction

As module identification depends highly on the constitution of the used PPI network, for a more robust module identification, we combined modules identified in three separate protein-protein interaction networks and computed their consensus modules (see above). The three networks were selected to find core network modules in unbiased, high confidence environment, which were extended with interaction data of various sources. We constructed the HI PPI network (4564 nodes and 15413 edges) by merging two PPI network of related studies published in ^14,15^ in which binary protein-protein interactions were systematically screened and validated through a well-established procedure resulting in a high quality and bias free representation of PPIs. The HI-Lit-BM (8153 nodes and 33392 edges) network consists of the HI network merged with the Lit-BM PPI network published in ^15^.The Lit-BM network was constructed using several public databases, which were filtered to contain only high confidence interaction. The MN network (13305 nodes and 138025 edges), published in ^16^, was constructed through combining several sources of protein interactions: (i) regulatory interactions derived from transcription factors binding to regulatory elements; (ii) binary interactions from several yeast two-hybrid high-throughput and literature-curated data sets; (iii) literature-curated interactions derived mostly from low-throughput experiments; (iv) metabolic enzyme-coupled interactions; (v) protein complexes; (vi) kinase-substrate pairs; and (vii) signaling interactions. We included this network in the analysis because of the wide range of interactions included in it. The overlap between nodes and edges in these networks is depicted in [**Figure S1**.](#Fig1)

# SECTION 2 – EXPANDED RESULTS

## Training-induced adaptations

The training-induced modules (**Figure 4**) were named after significantly enriched GO terms in the modules, detailed in **Additional file 2:**  **Table S5**.

In COPD patients, the **Hippo pathway module** suggests abnormal training induced activation of skeletal muscle remodeling. The module contains four genes exhibiting transcription factor activity, from which VGLL, TEAD3, TEAD4 showed significant down-regulation. We observed a significant up-regulation of WWTR1 – the main transcription factor of the Hippo signaling cascade, exhibiting pro-proliferative effects when associating with TEAD and SMAD family co-factors^17^. It is of note that none of the module genes were differentially expressed in healthy muscle marking the specificity of this mechanism to COPD (**Figure S5**).

The Healthy-TE network modules indicated strong associations of training responses with bioenergetics changes and their joint regulation with other molecular functions. The **Amino acid biosynthesis module** showed significant enrichment of genes associated with amino acid biosynthetic processes (ASNS, CTH), as well as to cellular organization processes such as macromolecular complex assembly (SF3A3, CTH, DVL1). Moreover, we found significant enrichment of **Intracellular transport** genes (ACTC1, KLHL2) in the similarly named module, and further analysis of specific gene functions showed its relation to amino acid biosynthesis (BCAM) and skeletal myofibril assembly (ACTC1). In the **Epigenetic regulation of metabolic processes module**, we identified the significantly down-regulated SET Nuclear Proto-Oncogene, together with other genes enriched for negative regulation of metabolic processes (SET, UBTF, YAF2). Gene level functions suggest the relation of the module to chromatin and histone modifications (SET, PTMA, UBTF). Finally, the genes of **Oxidative phosphorylation module** showed enrichment for GO terms related to ATPase activity coupled ion transport as part of the oxidative phosphorylation pathway (ATP6V1G1, ATP6V1D, ATP6V1E2).

## Impact of FFMI on disease effect (DE) modules

The post-hoc analysis suggested stronger disease effects in low FFMI (COPDL-DE), for which we identified 4 network modules and 1,700 differential expressed genes (DEGs) (FDR < 0.05); compared to normal FFMI (COPDN-DE) COPD patients, which indicated 2 network modules and 950 DEGs (see **Additional file 2:** **Table S1**). In COPDN, the strongest disease effects were seen in creatine metabolism and cell cycle organization; whereas, network module abnormalities in COPDL were most evident on Ca2+ dependent protein binding, cell morphogenesis, transcriptional regulation and disturbed regulation of muscular oxidative capacity signaled by the alteration in the steroid hormone receptor activity pathway (PPAR pathway) (see [**Figure S2**](#Fig2)).

## Impact of FFMI on training effect (TE) modules

The post-hoc analysis indicated both stronger and different training responses at gene level in COPD_N_ (COPDN-TE), for whom we identified 1,352 DEGs (FDR < 0.05), as compared to COPD_L_ (COPDL-TE) showing only 601 DEGs (see **Additional file 2:** **Table S1**). Similarly to the COPD-TE analysis (**Figure 3**), in COPD_N_-TE, we observed both Hippo pathway and Interferon response modules, which were complemented with modules indicating clear training-induced bioenergetics adaptations. In contrast, COPD_L_-TE did not show training-induced bioenergetics responses, but only muscle remodeling and hypoxia-related modules were found. Differences between the training induced regulation of genes in two COPD subgroups were also clearly identifiable at gene level (see **Figure S3**).

## Training effect differences between COPD and healthy

The analysis was aimed to identify network modules, representing the difference between the training effects of COPD and healthy muscle (ΔTE) ([**Figure S4**](#Fig5)**A**). Hippo signaling, over being one of the main training effect modules for COPD, also showed a significantly different training response in healthy and COPD muscle. The Histone acetylation module contained ING5 and JADE2 genes related to histone modification and acetylation ([**Figure S4**](#Fig5)**B**).

## Gender effect on the transcriptomics profile

Gender specific differences in the gene expression profiles were analyzed using principal component analysis ([**Figure S5A**](#Fig6)) and hierarchical clustering ([**Figure S5B**](#Fig6)). The mentioned methodologies were used to identify outlier samples in the transcriptomics data. In the analysis both before- and after-training measurements were included but only those gene’s gene expression profile was considered that was found differentially expressed in either the disease effect or training effect conditions in COPD (COPD-DE, COPD-TE) (n=3484). The first two principal components (PC) were considered in the analysis that explained 32% of the genes’ overall variability.

The ID of the two female participants were HC_10_BT/AT and HC_12_BT/AT, which did not show outlier behavior in either of the analysis. It is of note however that the before-training sample of the HC_12 female subject appeared in both analysis to be more similar to the after training samples, especially to its own after training sample. This could signal that the subject was relatively trained already at the start of the study.

# ONLINE SUPPLEMENT REFERENCES

1. Borg, G. A. Psychophysical bases of perceived exertion. *Med. Sci. Sports Exerc.* **14,** 377–81 (1982).

2. Nici, L. *et al.* American Thoracic Society/European Respiratory Society Statement on Pulmonary Rehabilitation. *Am. J. Respir. Crit. Care Med.* **173,** 1390–1413 (2006).

3. Troosters, T., Casaburi, R., Gosselink, R. & Decramer, M. Pulmonary rehabilitation in chronic obstructive pulmonary disease. *American Journal of Respiratory and Critical Care Medicine* **172,** 19–38 (2005).

4. Barabási, A.-L., Gulbahce, N. & Loscalzo, J. Network medicine: a network-based approach to human disease. *Nat. Rev. Genet.* **12,** 56–68 (2011).

5. Ideker, T., Ozier, O., Schwikowski, B. & Siegel, A. F. Discovering regulatory and signalling circuits in molecular interaction networks. *Bioinformatics* **18,** S233–S240 (2002).

6. Mitra, K., Carvunis, A.-R., Ramesh, S. K. & Ideker, T. Integrative approaches for finding modular structure in biological networks. *Nat. Rev. Genet.* **14,** 719–32 (2013).

7. Sharma, A. *et al.* A disease module in the interactome explains disease heterogeneity, drug response and captures novel pathways and genes in asthma. *Hum. Mol. Genet.* **24,** 3005–20 (2015).

8. Diez, D., Agustí, A. & Wheelock, C. E. Network Analysis in the Investigation of Chronic Respiratory Diseases. From Basics to Application. *Am. J. Respir. Crit. Care Med.* **190,** 981–988 (2014).

9. Leiserson, M. D. M. *et al.* Pan-cancer network analysis identifies combinations of rare somatic mutations across pathways and protein complexes. *Nat. Genet.* **47,** 106–114 (2014).

10. The Cancer Genome Atlas Research Network. Comprehensive molecular characterization of clear cell renal cell carcinoma. *Nature* **499,** 43–9 (2013).

11. Linehan, W. M. *et al.* Comprehensive Molecular Characterization of Papillary Renal-Cell Carcinoma. *N. Engl. J. Med.* (2015). doi:10.1056/NEJMoa1505917

12. Vandin, F., Clay, P., Upfal, E. & Raphael, B. J. Discovery of mutated subnetworks associated with clinical data in cancer. *Pac. Symp. Biocomput.* 55–66 (2012).

13. Faith, J. J. *et al.* Large-scale mapping and validation of Escherichia coli transcriptional regulation from a compendium of expression profiles. *PLoS Biol.* **5,** e8 (2007).

14. Rual, J.-F. *et al.* Towards a proteome-scale map of the human protein-protein interaction network. *Nature* **437,** 1173–8 (2005).

15. Rolland, T. *et al.* A Proteome-Scale Map of the Human Interactome Network. *Cell* **159,** 1212–1226 (2014).

16. Menche, J. *et al.* Disease networks. Uncovering disease-disease relationships through the incomplete interactome. *Science* **347,** 1257601 (2015).

17. Watt, K. I. *et al.* The Hippo pathway effector YAP is a critical regulator of skeletal muscle fibre size. *Nat. Commun.* **6,** 6048 (2015).

# SUPPLEMENTARY FIGURES


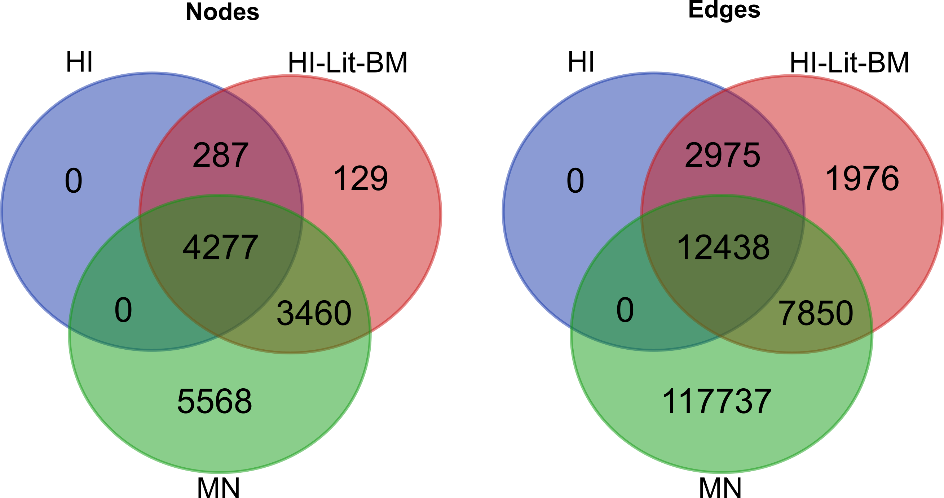


### **Figure S1.** Overlap between nodes and edges in the HI, HI-Lit-BM and MN interaction networks.

**
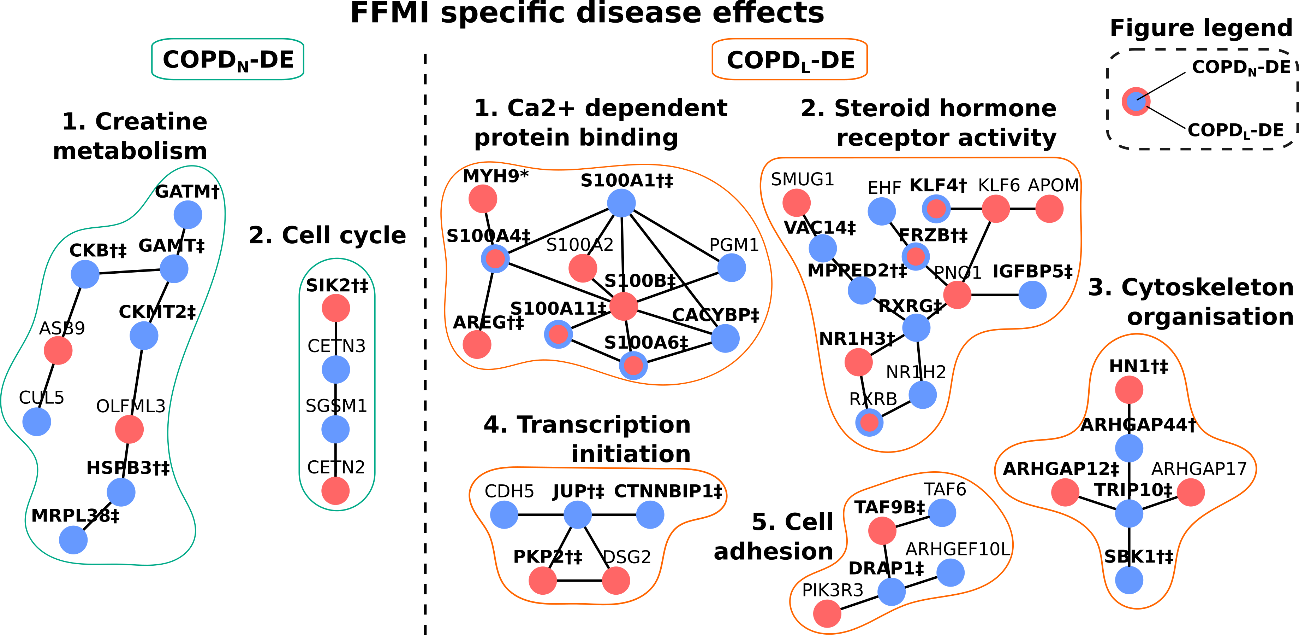
**

**Figure S2.** **Disease effect modules of the COPD subgroups.** The figure depicts functional modules associated to normal FFMI training effects (COPD_N_-DE) (on the left side with cyan borders) and low FFMI training effects (COPD_L_-DE) (on the right with orange border). Genes are colored according to their differential regulation in COPD_N_-DE (inner color of the nodes) and in COPD_L_-DE (border color of the nodes): blue color means down-regulation, red color means up-regulation and significant genes are signed by † in COPD_N_-DE and ‡ in COPD_L_-DE.

**
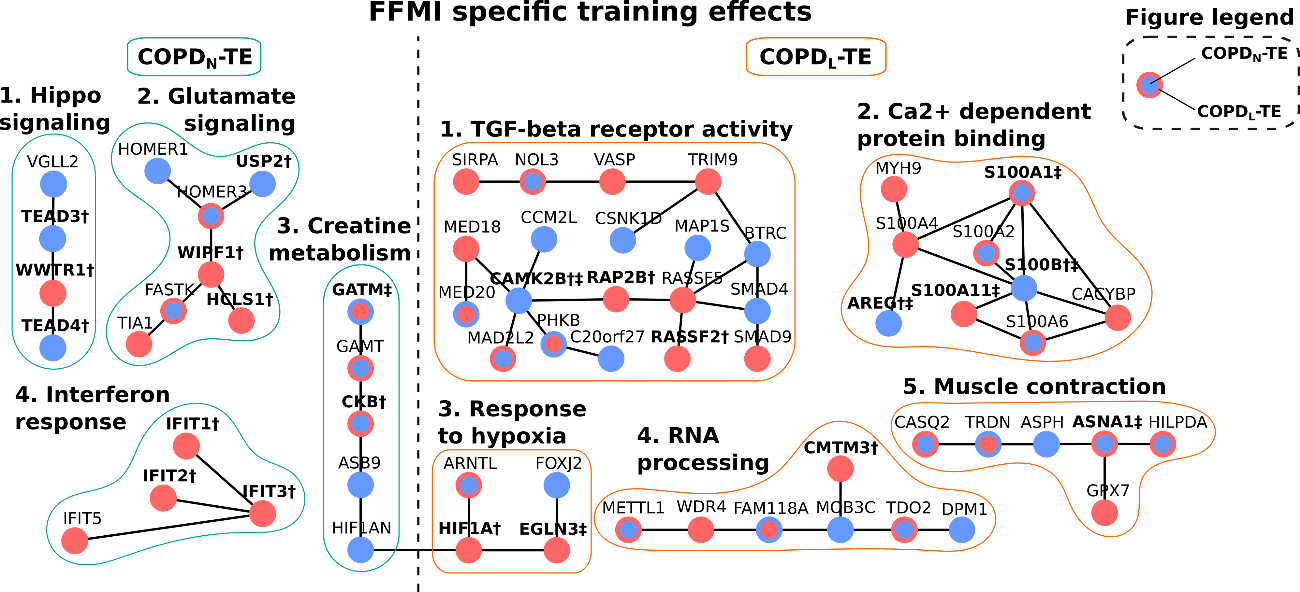
**

**Figure S3. Training effect modules of the COPD subgroups.** The figure depicts functional modules associated to normal FFMI training effects (COPD_N_-TE) (on the left side with cyan borders) and low FFMI training effects (COPD_L_-TE) (on the right with orange border). Genes are colored according to their differential regulation in COPD_N_-TE (inner color of the nodes) and in COPD_L_-TE (border color of the nodes): blue color means down-regulation, red color means up-regulation and significant genes are indicated by † in COPD_N_ -TE and ‡ in COPD_L_-TE.


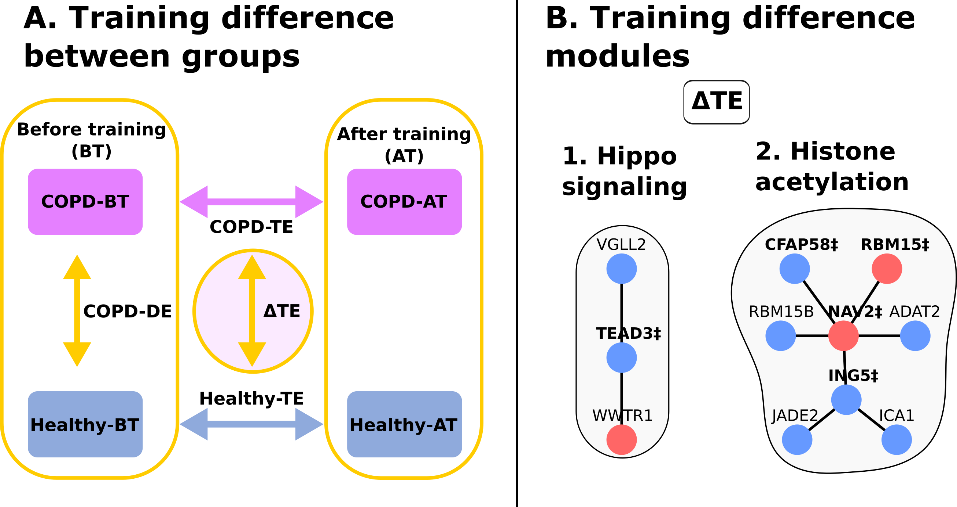


**Figure S4. Difference between COPD and healthy training effects.** (A) The analysis was aimed to identify network modules, representing the difference between the training effects of COPD and healthy muscle (ΔTE). Panel (B) depicts the two identified and functionally characterized network modules. Blue color means lower, red color means greater transcriptional change in COPD compared to healthy training effects. Significant genes are indicated by ‡.

**
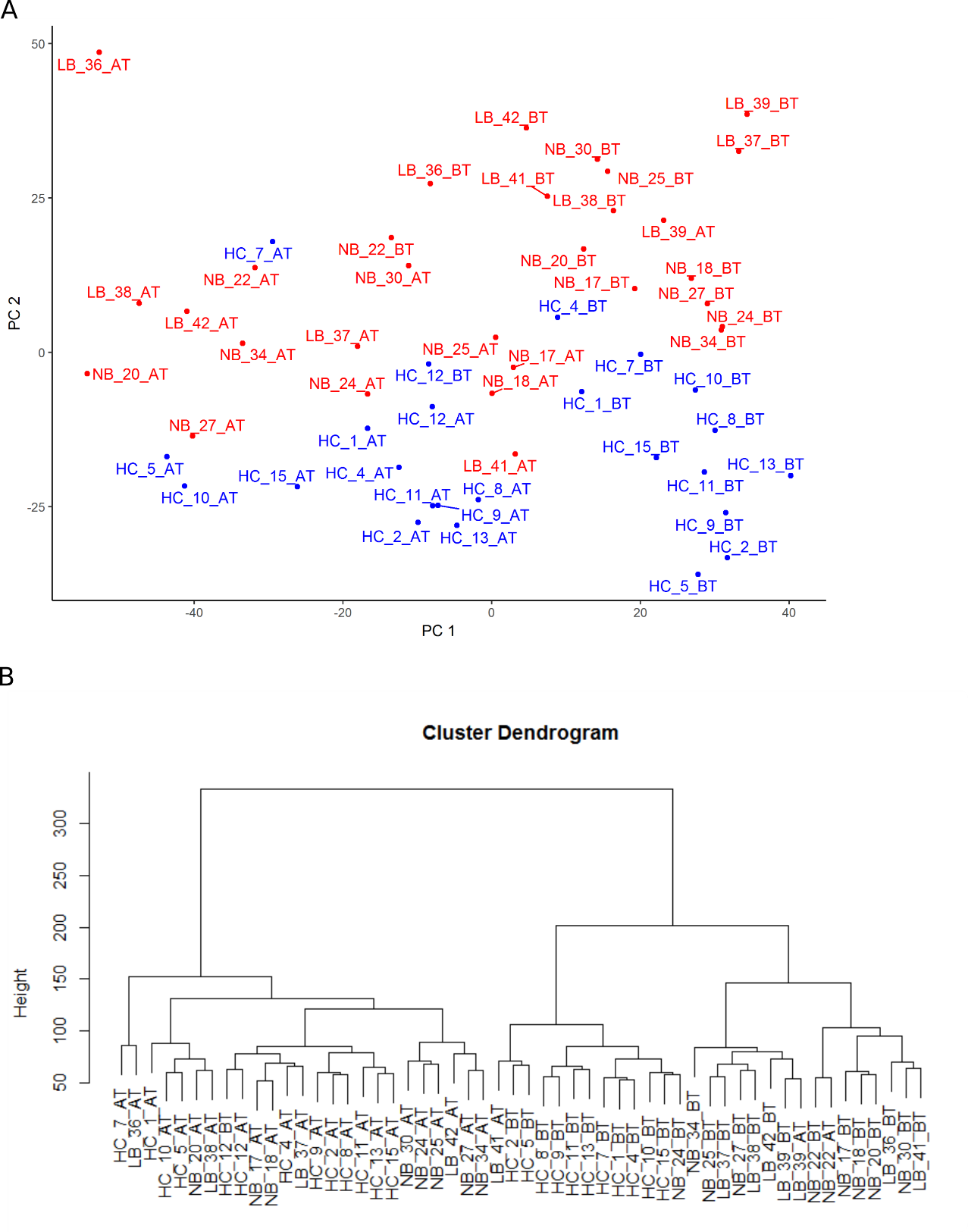
**

**Figure S5. Gender effect on the transcriptomics profile.** (A) Principal component analysis of the gene expression samples. (B) Hierarchical clustering analysis of the gene expression samples. In the analysis both before- and after-training measurements were included but only those gene’s gene expression profile was considered that was found differentially expressed in either the disease effect or training effect conditions in COPD (COPD-DE, COPD-TE) (n=3484). In both panel, labels of the samples indicate first the group (HC – healthy control, NB – normal body mass COPD, LB – low body mass COPD), then the sample identifier and finally the sampling time (BT – before training, AT – after training) separated by underscore characters. The ID of the two female participants were HC_10_BT/AT and HC_12_BT/AT.
